# Incidental findings of ectopic adrenal cortical tissue in the umbilical hernia sac and uterine wall: report of two cases in adult females

**DOI:** 10.1093/jscr/rjaf316

**Published:** 2025-05-17

**Authors:** Reza Khorvash, Aileen Azari-Yam

**Affiliations:** Arizona College of Osteopathic Medicine, Midwestern University, 19555 N 59th Ave. Glendale, AZ 85308, United States; Pathology Department, Medipol International University, Kavacık, Atatürk Cd. No: 40, 34810 Beykoz, Istanbul, Turkey

**Keywords:** ectopic adrenal tissue, adrenal rest, umbilical hernia, uterine serosa, incidental finding, pathology

## Abstract

Ectopic adrenal cortex is rare and usually found incidentally along the gestational migration path of the urogenital organs. It is rare in adult females, and most reported cases have been in male children. We report two cases of incidental ectopic adrenal cortex in 35- and 72-year-old women found in the hernia sac and uterine serosa, respectively. Histopathological and immunohistochemical analysis confirmed the diagnosis in both cases. Awareness of ectopic adrenal tissue is essential to avoid misdiagnosis, particularly in oncology patients where it may be mistaken for metastatic disease. Routine pathological examination of excised tissues can enhance early recognition and improve diagnostic accuracy.

## Introduction

The adrenal glands are normally located above the kidneys as retroperitoneal organs but have a different embryological origin from the kidneys [1, 2]. Their main role is to produce hormones such as cortisol, aldosterone, androgens, and catecholamines. It is postulated that during embryogenesis, the abnormal migration of adrenocortical primordial cells leads to the aberrant location of adrenal tissue. Ectopic adrenal tissues (EAT) are most commonly found along the migration pathway of the urogenital system, but they can also be located at sites such as the level of the celiac axis, spinal cord, broad ligament, and other retroperitoneal organs [3]. As the ectopic location is restricted to the cortical part and not accompanied by the medulla, it is referred to as adrenal cortical ectopy [4].

It is estimated that EAT is present in approximately 50% of newborns and usually disappears through atrophy within a few years [5, 6]. However, it may persist as either functioning or nonfunctioning tissue in less than 1% of individuals into adulthood [2]. It can be found in locations such as the kidney, retroperitoneal fat, testis, spermatic cord, ovary, liver, colon, gastric wall, and within the wall of the salpinx [4, 7, 8]. EAT has also been reported in up to 23% of surgical specimens of broad ligaments in total hysterectomy [9]. It is generally asymptomatic and often observed incidentally as bright-yellow nodules. It rarely becomes significant as a hormone-producing organ [2, 4, 9, 10]. If left untreated, these conditions can lead to mortality and morbidity.

Most reports of EAT are found in male children [6, 11], with the genitourinary tract being the most frequent location. However, it is rarely discovered in other locations and is often found incidentally [12, 13] during surgical procedures in the inguinal region of young infants and children [5]. Radiological diagnosis can be challenging, and it is usually not detected prior to surgery. Although its clinical significance is not well understood, EAT generally tends to be a benign condition.

In this case series, we present two unusual and incidental findings of EAT in the hernia sac of a female during elective abdominoplasty and umbilical hernia repair, which is even more uncommon, and review the literature. In the second case, EAT was found during the pathological examination of a hysterectomy specimen. This series aims to highlight these rare lesions and raise awareness of this entity and the potential diagnostic dilemmas due to their morphological similarities with other lesions, such as metastatic lesions, thereby improving understanding of this disorder. It also emphasizes the importance of sending all removed tissues for pathological evaluation.

## Case 1

**Figure 1:**
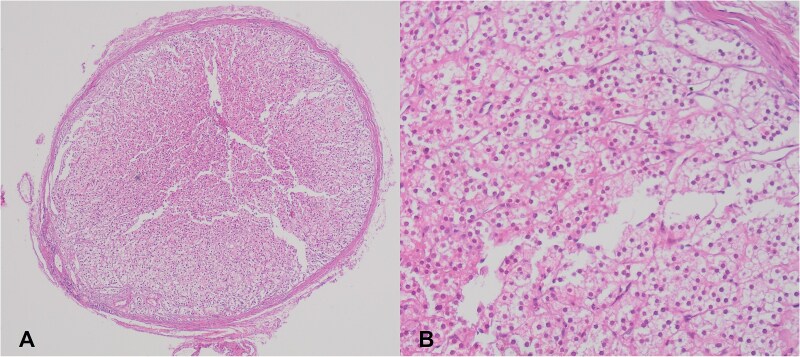
(A) Adrenal cortical ectopic tissue was found incidentally as a well-demarcated nodule during herniorrhaphy in a 35-year-old woman. (B) Microscopic view of the same nodule showing clear to eosinophilic epithelial cells arranged in cords and nests.

A 35-year-old woman underwent elective surgery for umbilical hernia repair along with cosmetic abdominoplasty and liposuction. Her medical history included two pregnancies, both of which were uncomplicated normal vaginal deliveries. Preoperative abdominal ultrasound and routine blood work were within normal limits. After completing the liposuction, during the herniorrhaphy, the surgeon discovered a small yellowish-tan nodule measuring 4 × 4 mm on the apex of the hernia sac (Fig. 1A). After liposuction, the abdominal flap was elevated with deeper dissection over the rectus abdominis aponeurosis. Then, the umbilicus was freed, and diastasis repair was performed. Afterward, the repair of the umbilical hernia was successfully completed. The nodule within the hernia sac was resected and sent for pathological examination. Following this, the abdominoplasty was performed. The patient was discharged without complications after a 1-day stay in the hospital. Pathology results indicated the presence of an encapsulated nodule (Fig. 1A) consisting adrenal cortical tissue within the hernia sac, composed of clear to eosinophilic epithelial cells arranged in cords and nests with intervening sinusoidal spaces, suggesting a diagnosis of EAT (Fig. 1B). Postoperative endocrine assessment indicated normal hormone levels.

## Case 2

A 72-year-old woman with uterine prolapse underwent an elective hysterectomy. She had a history of four pregnancies and experienced menopause at the age of 55. Her physical exam, ultrasound, and blood work revealed were unremarkable. Macroscopic examination of the uterus revealed no abnormal finding. The patient was discharged following the procedure. Histomorphologic examination showed a small well-circumscribed nodule within the uterine serosa. Histologically, the nodule was surrounded by a thin fibrous capsule and was composed of small cords and trabeculae of polygonal cells with distinct cellular borders. The cells had small and uniform nuclei without atypia or mitoses. The cytoplasm appeared clear to eosinophilic. In some foci the cells were filled with lipid-rich vacuoles. The pattern of cellular configuration was similar to adrenocortical tissue, suggesting EAT, while the uterus and ovaries showed age-related changes. No adrenal medulla was found. A postoperative endocrine evaluation revealed normal hormonal levels.

## Discussion

In this study, we reported two ETA found in unusual locations in two healthy adult females. A review of the English literature disclosed fewer than 150 reported cases of ETAs, supporting the theory that adrenal ectopia is rare but not exceptionally uncommon, as suggested by other case series. The adrenal cortex is susceptible to developing ectopic tissue [5]. Ectopic adrenal tissue can present as either a heterotopic adrenal gland or an accessory adrenal gland. Heterotopia occurs when adrenal tissue migrates abnormally during early embryogenesis, leading to its presence in adjacent organs such as the kidneys and liver. In contrast, an accessory adrenal gland forms due to abnormal migration of adrenal tissue during the later stages of development. In both cases, only the adrenal cortex is typically found at the ectopic site without the medullary component [14].

Ectopic adrenal tissue can be located in the retroperitoneum at various sites, ranging from the diaphragm to the pelvis, but it is usually found along its path of descent or in association with the gonads [15]. This condition is most commonly reported during childhood, particularly in relation to the genitourinary system in male children [8]. However, the presence of adrenal tissue in the umbilical sac and uterine wall in older females is extremely rare. This rarity may be due to the atrophy of adrenal tissue over time [16].

The presence of adrenal cortical tissue adjacent to the gonads or along their pathway of descent is expected due to the close spatial relationship between the adrenal primordium and the genital ridge during early embryonic development [7]. However, the occurrence of ectopic tissue in an umbilical hernia sac or adjacent to the uterus is more difficult to explain. One possibility is that adrenal cortical cell aggregates become enclosed within the peritoneum and subsequently migrate along with adjacent organs to their final locations, such as the umbilicus, uterus, or even other aberrant sites, including intracranial locations [17]. A mechanism of primitive cell entrapment has previously been proposed for splenogonadal fusion, and this model may also explain the unusual and aberrant localization of ectopic adrenal tissue [18].

Histopathologic examination is the gold standard way for its diagnosis. The main differential diagnosis of this condition is metastatic carcinoma especially clear cell carcinomas such as renal cell or hepatocellular carcinoma that could be ruled out by using immunohistochemistry in suspicious cars although it seldom is necessary.

It is crucial for surgeons to be aware of such lesions, especially in unusual locations beyond the typical sites, such as the spermatic cord. Furthermore, ectopic adrenal cortical tissue may be associated with hyperplasia or neoplastic processes [9]. It has also been proposed that the presence of ectopic adrenal tissue in the inguinal region is related to hernial sacs [5]. Also, adrenal rests have been reported in 23% of broad ligament specimens removed during total hysterectomy [19].

Abdominoplasty and pelvic organ prolapse repair are common plastic and gynecologic surgical procedures [20, 21] which provide both cosmetic and functional benefits. Abdominoplasty is especially fruitful after significant weight loss [20, 22], while herniorrhaphy improves quality of life, particularly in older women [21, 23]. During fetal development, the fascial opening or umbilical ring at the base of the umbilicus serves as a passage for umbilical vessels that connect mother and fetus. After birth, this opening gradually closes as the rectus abdominis muscles are displaced toward each other. By the age of five, complete fusion of the peritoneal and fascial layers occurs in approximately 85% to 90% of children [24]. Many women experience umbilical hernias after pregnancy, as the umbilicus serves as a site for herniation due to a lack of muscular support and resultant weakness in the surrounding tissue [20, 22]. By concurrent umbilical hernia repair during abdominoplasty, the need for a separate surgical procedure is eliminated [20, 25]. The contents of the hernia sac are removed during herniorrhaphy and the fascial defect is repaired. Pelvic organ prolapse is also a common condition that significantly impacts the quality of life. Surgical management is usually offered when noninvasive and supportive measures have failed [2, 26]. The surgical approach may be vaginal or abdominal, depending on the patient’s condition and accompanying pathologies [27].

Here, we reported two elective surgeries with no complications, and both of our reported patients were followed up for 2 years with no symptoms or signs of abnormal adrenocortical hormone production or imbalance. The finding of adrenal rests in these two benign conditions was unexpected. These could be alarming at the time of surgery, as they may grossly mimic a cancerous implant. Furthermore, they could produce excessive hormones and become tumorous in the same way as native adrenal glands.

During the evaluation of cancerous patients, these ectopic tissues can be misinterpreted as metastatic deposits. If they are detected only radiologically or at the time of surgery without a frozen section diagnosis, they can lead to changes in surgical and therapeutic approaches, potentially resulting in overtreatment and overstaging of these patients. If they become hyperplastic and functional, they may cause hormonal imbalance with an occult primary disease. This also emphasizes the prior statement from the American College of Surgeons that “all tissues removed at operation shall be examined and reports rendered thereon” [28]. This was also declared in 1998 by the Joint Commission on Accreditation of Healthcare Organizations, which stated that “specimens removed during surgery need to be evaluated for gross and microscopic abnormalities before a final diagnosis can be made” [28, 29]. The necessity of pathological examination of herniorrhaphy specimens remains controversial [28, 30, 31]; however, this study highlights the importance of thorough examination to ensure appropriate patient care despite pressures in order to maintain cost-effective practices.

In conclusion, the ectopic adrenal cortex typically atrophies over time and is rarely found in adults, especially in females. While ectopic adrenal tissue is usually asymptomatic and nonfunctional, its recognition is important. Functional ectopic tissue can cause clinical issues. Accurate identification is essential, especially in patients with a cancer history, to guide proper surgical planning and prevent misdiagnosis of metastasis. Routine submitting of all excised tissue for pathological examination is important, and documenting such rarities can enhance awareness among surgeons and pathologists, resulting in a better patient understanding of the pathogenesis underlying its occurrence.
