# Correction to: Incidental findings of ectopic adrenal cortical tissue in the umbilical hernia sac and uterine wall: report of two cases in adult females

**DOI:** 10.1093/jscr/rjag377

**Published:** 2026-05-15

This is a correction to: Reza Khorvash, Aileen Azari-Yam, Incidental findings of ectopic adrenal cortical tissue in the umbilical hernia sac and uterine wall: report of two cases in adult females, *Journal of Surgical Case Reports*, Volume 2025, Issue 5, May 2025, rjaf316, https://doi.org/10.1093/jscr/rjaf316

The following change has been made to the originally published paper. The authors have been transposed in order in the author list.

